# Promising Practices in Capacity Development for Health Supply Chains in Resource-Constrained Countries

**DOI:** 10.9745/GHSP-D-23-00208

**Published:** 2025-05-09

**Authors:** Mahama Duwiejua, Pamela Steele, Paul Lalvani, Dorothy Leab, Lloyd Matowe, Jonathan Moody

**Affiliations:** a Pharmaceutical Systems Africa, Accra, Ghana.; b Pamela Steele Associates Ltd., Nairobi, Kenya.; c Empower School of Health, Haryana, India.; d GaneshAID, Hanoi, Vietnam.; e Manica University, Lusaka, Zambia.; f People that Deliver, Copenhagen, Denmark.

## Abstract

Performance gaps in health supply chains in low- and middle-income countries contribute significantly to inefficiencies and underperformance of their health systems. Some significant factors hindering the performance of supply chains in low and middle-income countries include low human resource capacity and capability, weak structures for monitoring supply chain performance, weak in-service and pre-service training programs, chronic underfunding, lack of transparency and an overdependence on obsolete methods, like manual data gathering, record-keeping, and analyses. Although proven health supply chain strengthening techniques exist, the level of adoption of these practices has varied across countries, resulting in multiple capacity gaps and underperforming supply chains. The resulting challenges require complementary and needs-based practices to address the gaps. While it is recognized that there is no “one-size-fits-all” solution to these issues, we demonstrate that real benefits can be achieved by using “promising practices”—that is, using targeted, innovative interventions. To demonstrate the potential of using promising practices in the health supply chain in Africa and the breadth of possible solutions available, we present 3 case studies from different contexts and with different objectives.

## Abstract (precis)

We present 3 country cases with varied objectives to illustrate the potential of innovative, promising practices as potential solutions for strengthening supply chains in low- and middle-income countries.

## Teaser

**Key Messages:**

- Suboptimal supply chain management/functions result in avoidable shortages and health commodity wastage as a consequence of a lack of real-time reliable data visibility and inappropriate quantifications and expiries.
- Low- and middle-income countries (LMICs) are making substantial progress toward solving their supply chain challenges through creative adoption of existing tools and by implementing promising practices by collaborating with development partners to build capacity and resilient supply chains.
- The case studies presented illustrate how challenges of weak staff capacity/capability, overdependence on obsolete electronic logistics management information systems, and absence of eLearning platforms with flexibility for use by busy non-core supply chain practitioners were addressed.

**Key Implications:**

- Sustainability and acceptability of promising practices to strengthen supply chains require stakeholder engagement and the adoption of needs-based solutions.
- Complexity of electronic logistics management information systems platforms should be appropriate for the level of users. The systems adopted should promote accountability, transparency, and sustainability.
- Training modules must be flexible enough to accommodate the schedules of busy non-core supply chain practitioners.

## INTRODUCTION

The availability of health commodities (medicines, vaccines, and other health products) is pivotal to achieving Sustainable Development Goal 3.^1^^,^^2^ The uninterrupted availability of health commodities depends on well-functioning, robust supply chain systems. We define the health care supply chain as the network of systems, components, and processes that collectively work to ensure medicines and other health care supplies are manufactured, procured, distributed, and provided to patients at health service delivery points. Effective supply chain performance is, therefore, pivotal to the success of a health care delivery system, whether public, faith based, employer provided, or private. Most low- and middle-income countries (LMICs), in collaboration with multilateral aid agencies, use a combination of proven practices (interventions with proven outcomes in improving health commodity supply chains in LMICs)^3^ and promising practices (interventions showing progress toward improving health commodity availability in supply chains in LMICs)^3^ to make significant improvements in their supply chains. Although progress has been made in many contexts, significant gaps remain in the capacity of LMICs to ensure the availability and delivery of health commodities at the right time, place, and cost. Consequently, health systems remain weak, rendering them incapable of responding optimally to the health care needs of the population.^3^^–^^6^ The need for strategies to strengthen the structural weaknesses and stabilize the performances of health supply chains, particularly in Africa, has been emphasized in several publications.^7^^–^^9^ Epidemics like Ebola and COVID-19 have exposed the vulnerability and lack of resilience of health supply chains in LMICs.^10^^,^^11^ Challenges requiring interventions range from (1) low capability maturity scores in strategic planning and management, with consequences such as duplication of efforts resulting from overlapping roles and responsibilities, lack of clarity of roles and authority, and lack of trust among actors within supply chains; (2) absence of supply chain risk management planning at institutional levels; (3) pervasive human resource capacity challenges; (4) weak in-service and pre-service training programs; (5) chronic underfunding of supply chains, unstable currency exchange rates, and frequent diversion of funds from drug revolving funds for unrelated activities like salary supplements; (6) overdependence on outmoded methods of data collection and management, like using manual forms owing to the absence of effective electronic logistics management information system (eLMIS) platforms to provide reliable real-time data and capacity to manage the data to inform policy decisions; and (7) absent or weak emergency supply chain protocols.^8^^–^^11^

Strategies for countries to address these challenges are varied and complex, requiring context-specific interventions. An in-depth understanding of a country’s supply chain management needs can be obtained through a gap analysis or supply chain process mapping. This exercise provides information on key stakeholders, their roles, and responsibilities in the supply chain and a capability maturity assessment on functional areas and cross-cutting elements like human resources, forecasting and planning, warehousing and storage, logistic management information systems, and financial sustainability. A useful tool for this exercise is the U.S. Agency for International (USAID) National Supply Chain Assessment 2.0.2 toolkit, which enables a holistic approach to analyzing country supply chains as opposed to traditional approaches that tend to focus on isolated processes within the supply chain.^12^ Other complementary effective tools for evaluating country supply chain information systems include USAID’s supply chain information system maturity model^13^ and the Interagency Supply Chain Group’s harmonization of key performance indicators.^14^

Strategies for countries to address supply chain challenges are varied and complex, requiring context-specific interventions.

Which practices have LMICs adopted to mitigate the challenges of underperforming supply chain systems? Furthermore, what lessons can be learned from the practices for scalability to cover the entire spectrum of supply chain activities in LMICs? In contrast with the Organization for Cooperation and Development countries, where the private sector has been the dominant driver for supply, distribution, and provision of key auxiliary supply chain services in the health system, in LMICs, the performance of supply chains in the public sector is crucial. This is primarily because, over the years, average product availability in public health facilities has been roughly 38%.^15^ Additionally, the public sector has been shown to lack the capacity to handle the increasing volume and complexity of donor support for health commodities.

Which practices have LMICs adopted to mitigate the challenges of underperforming supply chain systems?

In this article, we provide 3 examples from the public sector in Ghana and Ethiopia and the World Health Organization Regional Office for Africa (WHO/AFRO) on promising practices for strengthening supply chain infrastructure and performance in different contexts. Through these examples, we aim to show promising practices that have the potential for scalability to improve health product availability and, by implication, health outcomes in resource-constrained countries.

## CASE STUDY 1: STRENGTHENING THE CAPACITY OF ETHIOPIA’S SUPPLY CHAIN WORKFORCE

In 2018, to strengthen the capacity of the supply chain workforce, the Ethiopia Pharmaceutical Supply Service (EPSS), established to supply quality-assured and affordable pharmaceuticals to public health facilities, embarked on interventions to address capacity gaps in human resources and supply chain systems at all levels. The interventions aimed to enable national health care transformation for Ethiopia and achieve organizational change for EPSS by following the strategic direction outlined in the Health Sector Transformation Plan and Pharmaceutical Sector Transformation Plans I and II.

To further improve EPSS’s supply chain operations performance,^16^ the EPSS’s Admas Programme was launched with support from Pamela Steele Associates and its partners and funding from the Bill & Melinda Gates Foundation to strengthen supply chain and human resource directorates’ technical and behavioral capabilities.^17^ Partnering with a team of subject matter specialists, Pamela Steele Associates used People that Deliver’s Theory of Constraints analysis to reveal the limiting factors that hindered the achievement of the supply chain’s goals and systematically guide the implementation of interventions to address the constraints.

The team conducted an extensive literature review of key strategic and operational documents and evaluation reports, the findings of which were reviewed at a June 2019 validation workshop in Addis Ababa with EPSS’s 19 top management officials. During the validation workshop, the supply chain constraints were analyzed, discussed, and validated. At the end of the workshop, the expert panel suggested several ways to improve, including adopting the agile-based methodology; using a change commitment curve, process maps, and scorecards; and applying the plan-do-act-check for each bottleneck.^16^

The process revealed gaps such as weak workforce capability, a lack of performance measures and rewards, low motivation, poor organizational culture, and high staff turnover. In addition, traditional and poorly developed human resources management systems and practices that focused on routine personnel administrative tasks rather than playing a strategic role in the supply chain workforce continued to present a challenge. The validation exercise categorized most of the challenges into 3 areas: quantification and market-shaping, procurement and contract management, and warehousing and distribution.

To address these challenges, the Admas Programme implemented targeted interventions that improved supply chain operations performance and boosted staff capacity and capability. These interventions focused on improving staffing and onboarding, strengthening the supply chain skills of EPSS staff, and improving working conditions by providing on-the-job training and mentoring across all functions. The Admas Programme’s interventions enabled the system to achieve a silver performance level, defined as a process maturity score of between 60% and 79%, in the elementary supply chain maturity assessments, which was a significant improvement. These targeted interventions resulted in improved technical competencies in supply chain management, a more empowered workforce, improved processes, reduced supply lead times and costs, and improved supply chain maturity. EPSS’s tender lead time decreased by 5.5% from 107 days in 2018 to 103 days in 2020. According to unpublished EPSS monitoring and evaluation reports, from 2019 to 2020, EPSS’s procurement lead time decreased by 25% from 296 days to 223 days, and contract signing lead time decreased by 35% from 17 days to 11 days, respectively.

The Admas Programme implemented interventions to improve staffing and onboarding, strengthen the supply chain skills of EPSS staff, and improve working conditions.

The Admas Programme conducted a training needs assessment based on the competency framework, created a training plan, and trained the EPSS workforce on various supply chain management topics. A competency assessment showed consistent and significant improvement in all 19 competency areas. A critical success factor was EPSS staff taking responsibility and ownership of their supply chain operations and decisions, thereby reducing their dependency on technical expertise offered through the program. However, skills development alone is not enough and must be balanced with proper infrastructure strengthening, strong human resources management, performance management, and an enabling environment.

The approach embraced sustainability from the beginning through the ownership and support of top leadership, directors, and operational staff at all levels (headquarters in Addis Ababa and all branches). By the end of the intervention period, EPSS staff were able to lead their supply chain operations and skills development.

## CASE STUDY 2: IMPROVING SUPPLY CHAIN PERFORMANCE WITH THE GHANA INTEGRATED LOGISTICS SUPPLY CHAIN MANAGEMENT INFORMATION SYSTEM

In 2019, to leverage the success of the 2015–2020 Supply Chain Master Plan (SCMP),^18^ Ghana embarked on a process of assessing the SCMP to develop an improved and more efficient health commodity 2021-2025 SCMP.^19^ The Ghana Health Service and the Ministry of Health (MOH), in collaboration with USAID and Global Health Supply Chain-Procurement and Supply Chain Management, conducted a National Supply Chain Assessment^20^ focusing on 11 technical areas: strategic planning and management, policy and governance, human resources, financial sustainability, forecasting and supply planning, procurement and customs clearance, distribution, logistics management and information system, quality and pharmacovigilance, waste management, partnering with the private sector. The assessment concluded that the supply chain had several deficiencies across functions, levels, and MOH agencies and lacked data visibility, coordination, and alignment of incentives and objectives.

These gaps informed the organization of the 2021–2025 SCMP.^19^ A key component of the SCMP is the Ghana Integrated Logistics Management Information System (GhiLMIS), an eLMIS that promotes greater efficiency through a web-based platform that connects all supply chain functions, ensuring data timeliness, visibility, completeness, and accuracy. An eLMIS tracks products’ location and movements, the rate of consumption/use, stock levels throughout the system, risks of stock-outs or expiry, temperature excursions for cold chain equipment, asset functionality for cold chain or diagnostic equipment, and operational performance at all levels of the supply chain.^21^

Information for this case study was obtained from desk reviews of MOH documents and key informant interviews with the Director of Pharmaceutical Services of the MOH, the Director of Procurement & Supply Chain of the MOH, and a consultant at Systems for Development (the lead subject matter specialist for developing GhiLMIS and implementing partner for the Global Fund in Ghana). To verify the interoperability of the GhiLMIS with supply chain functions at peripheral institutions, visits were made to the warehouses of the Logistics Management Unit of the MOH and Imperial Health Service (private sector partner of the MOH), a teaching hospital, a regional medical store, and a regional hospital.

GhiLMIS has demonstrated potential as a sustainable, promising practice with accrued benefits to the system (unpublished data).

First, coverage improved significantly. By the end of the first quarter of 2023, GhiLMIS had expanded to more than 3,000 sites across all levels of the Ghana Public Health Commodity Supply value chain. Additionally, around 5,000 active users have been trained, onboarded, and equipped with the necessary skills to effectively use GhiLMIS to manage their commodities.

Uptake and utilization of GhiLMIS also increased to 80% by the first quarter of 2023 for at least 1 of the system modules. Purchase order is the widely leveraged functionality that effectively supports requisition planning and execution. The use of this has also positively affected the bimonthly distribution of commodities from the central to mid-tier and the respective region’s last-mile distribution commodity distribution to service delivery points from the mid-tier of the value chain.

Uptake and utilization of GHiLMIS increased to 80% by the first quarter of 2023 for at least one of the system modules.

Operational efficiencies were also seen. GhiLMIS has contributed to an overall decrease of 98% in transactional cycle time across all implemented sites. There has also been a reduction of overall inventory management and operational costs of US$2.6M per year, resulting in substantial savings at the central level. GhiLMIS has contributed to eliminating an estimated US$230K annually associated with the printing and dissemination of reports, requisition, issues, and receipt vouchers and reducing $380K annually associated with the transportation, per-diem, and transmission of orders from requesting sites (i.e., service delivery points, district health directorates) to the fulling sites (i.e., temporary central medical store and regional medical stores).

GhiLMIS has also provided the required platform for end-to-end visibility and availability of near real-time data across the value chain, facilitated the effective management of COVID-19 commodities, is currently being used as the supply chain platform for pandemic management, and has enabled established data standards and built a platform that is leveraged as the Single Version of Truth, facilitating quality transactional data capture, processing, and insights to support decision-making.

Key challenges to the sustainability of GhiLMIS include unreliable power supply and Internet connectivity, limited availability of hardware like computers, and limited technical training/knowledge at service delivery points coupled with the excess workload of providers, making the conversion from traditional paper-based systems, with its inefficiencies, difficult.

## CASE STUDY 3: USING A MOBILE LEARNING TOOL TO BUILD SUPPLY CHAIN COMPETENCY IN AFRICA

Continuous professional development for health workers in immunization supply chain management is key for ensuring the uninterrupted availability of potent vaccines, appropriate usage, and monitoring of immunization programs. The need to systematically and continuously strengthen the performance of immunization supply chain (ISC) professionals in Africa is hampered by challenges, including costly in-person training courses that cannot address all individual training needs; the long duration of eLearning courses with a high learner dropout rate; difficulty in accessing up-to-date reference materials scattered over multiple websites; insufficient community of practice for peer-to-peer learning and support; and absence of a tool to monitor ISC staff performance.

WHO/AFRO provided a solution to these challenges by introducing an innovative and adaptive mobile social learning tool, AFRiSC, to reinforce the capacity and competencies of cold chain and logistics staff and managers operating and managing the ISC systems in different WHO/AFRO countries.^22^ To address the challenges confronting busy non-core supply chain operators as well as supply chain professionals in resource-constrained environments, AFRiSC’s platform comprises functionalities including:

- Individual learning pathways for key ISC positions (logistician, cold chain officer, storekeeper, ISC manager, in-country partner, and national logistics working group
- Micro-learning capsules for bite-sized learning accessible anytime online and offline
- Knowledge library with up-to-date reference documents
- Africa ISC social network with a personal page, forums, and instant messaging features to connect with peers.

These functions are expected to lead to improved ISC staff competencies and an empowered workforce at all levels, including improved bidirectional communication between WHO and in-country ISC staff, the creation of a network of ISC staff among the 47 AFRO member states for a stronger ISC culture, peer learning and sharing, and improved capacities for WHO to monitor the learning and performance of ISC staff within the 47 member states.

AFRiSC’s functions are expected to lead to improved ISC staff competencies and an empowered workforce at all levels.

The viability of this intervention is high as it has been co-designed with users to ensure the suitability of the digital solution. The WHO/AFRO team is trained to own and manage the mobile app administration platform. Users’ activities are tracked on the platform for easy monitoring and learning. Most importantly, the solution is adaptable depending on users’ needs and demands.

WHO has defined a strategy to scale up this intervention. The first phase will target Francophone and Anglophone countries in Africa. In its second phase, AFRiSC will also be made available in Spanish and Portuguese. AFRiSC is free of charge and can be institutionalized in some countries as part of the national master training plan for ISC personnel.

In its third phase, AFRiSC could be expanded to other WHO regions and integrate other essential health programs in the framework of primary health care. The application provides targeted training for non-core supply chain professionals outside working hours and offers recorded lectures that can be accessed at the leisure of the health worker. The adoption of this platform is promising and sustainable; it is built and led by local expertise and has demonstrated value to justify future investments. The implementation approach has involved critical stakeholders from inception through all implementation phases to provide specific solutions to address the supply chain needs of countries. Most importantly, there is political support for this mobile and social learning program.

## DISCUSSION AND RECOMMENDATIONS

The supply chain, as Yadav described,^23^ is not simply a set of warehouses, trucks, and carton boxes but rather comprises the ecosystem of organizations, people, technology, activities, information, and resources that have to come together to ensure delivery of the product from the point where it is manufactured to the end-user, in a cost-effective way. A functional supply chain must have structures at all operational levels to include features that return critical information regarding need, demand, and consumption to health system planners. It is, therefore, a complex system of operations requiring significant levels of expertise. While there is no “one size fits all” approach, the different methods described in the case studies demonstrate that major improvements in supply chain management can be made by accurately diagnosing challenges and adopting and implementing targeted interventions.

The case of the EPSS illustrates gains that can be made through capacity-building of supply chain staff at 1 node of the chain—the center. When systems are weak, a holistic approach to building capacity across supply chain functions yields improvements in the system, from the central level to service delivery points. However, owing to resource constraints, it is not always financially prudent to carry out a complete assessment of a functional supply chain to determine weak points for interventions. Therefore, supply chain managers are encouraged to be proactive and adopt strategies that deploy targeted interventions for improved performance.

Reliable data availability and analyses are the backbone of a functional supply chain. The case of the GhiLMIS demonstrates gains that accrue from introducing an eLMIS to reduce overreliance on outmoded methods of data collection and management. Deploying GhiLMIS is a promising intervention with the potential to affect all functional areas of the country’s public health supply chain to make the system more efficient. In LMICs with weak stock management capabilities, particularly at the facility level, the push system is often used with all its associated limitations. The adoption of an eLMIS provides the infrastructure for rational decision-making based on information flow from the lowest to the highest levels of the health systems. GhiILMIS has features that allow interoperability with other supply chain systems in the public health sector and national health information systems containing vital data streams such as disease prevalence and health facility data. Thus, the problem of standalone solutions is reduced, and coordination and collaboration of functions within the supply chain system are improved. A lack of transparency in supply chain practices exposes the process to interference from external forces, fostering corruption and inefficiencies. By promoting end-to-end data visibility and analytics across all supply chain levels using real- or near-real-time quality transactional data, GhiILMIS promotes transparency. The quality assurance of products is an equally important function that must be incorporated into supply chain processes, especially increasing the ability to detect falsified and substandard drugs in the system. An assessment of counterfeit reports involving the legitimate supply chain using 2009–2011 data from the Pharmaceutical Security Institute Counterfeit Incident System database indicates the scale of infiltration of counterfeit medicines into legitimate supply chains to be huge and not limited to only LMICs.^24^

Creating an electronic platform does not automatically translate into gains for the system. A major difficulty is the acceptance of the system by non-core supply chain professionals with clinical responsibilities. These health care professionals, already suffering from work overload and burnout, find it difficult to build capacity for the new system. AFRiSC, the innovative and adaptive mobile social learning tool developed by WHO/AFRO, is an important solution for these busy non-core supply chain professionals performing supply chain functions. The software offers flexible self-learning opportunities and peer learning. Scale-up and acceptability by users can be enhanced with the institutionalization of supportive and mentoring structures. Konduri et al.^25^ demonstrated the value of supportive structures and mentoring in their study on the adaptation and deployment of various training modalities to meet country-specific contexts and needs. This study involved a global pharmaceutical system strengthening program in collaboration with a country’s MOH and local stakeholders.

Acknowledging the unique challenges and specific needs of non-core supply chain managers and practitioners at remote facilities, a streamlined version of GhiLMIS has been developed to enhance its usability. This version is customized for the needs of lower facility managers, devoid of complex functionalities relevant to higher decision-makers.

GhiLMIS is built on a platform/philosophy similar to that of OpenLMIS,^26^ a leading open-source or public domain eLMIS purpose-built to manage health supply chains in LMICs. More than 11,000 health facilities in 9 countries across Africa, across all major health programs including vaccines and HIV/AIDs, have adopted public domain Internet-enabled LMIS. Like GhiLMIS, OpenLMIS has proven to reduce stock-out rates, save health workers’ time, and improve data visibility.^26^ For acceptance, an eLMIS must fit the local environment and be scalable to accommodate future growth. Interoperability with existing information systems (e.g., warehouse management, medical records, laboratory management, enterprise resource planning systems, and health information management systems) is critical for acceptance.

Initiating supply chain reform is a delicate and complicated endeavor. Solutions are context specific. Thus, in the absence of data driven by well-controlled scientific evaluations and analysis, interventions from different contexts should be adopted with caution because external validation of the evidence is weak. For these reasons, the practices we documented are labeled as promising in contrast with proven practices that are more scientifically validated. The cases presented have proven relevance and promise for scalability. To promote efficiency in supply chain functions in resource-constrained environments, the scale-up activities should extend beyond public health institutions to include faith-based and private-sector health providers. For example, the Christian Health Association of Ghana, which delivers 30%–40% of health services, is a major implementing partner of the MOH and operates within the MOH’s policies, guidelines, and strategies, so the GhiLMIS operations are extended to the association’s facilities.

We reiterate that supply chain management is a management science that requires expertise to ensure the availability of vaccines, medicines, and other lifesaving health technologies when and where they are needed. Ineffective and poorly designed supply chains for health commodities have synergistic effects with health workforce shortages, creating barriers to achieving the sustainable development goal of good health and well-being for all.

The cases presented support the calls for innovative solutions to the challenges of underperforming supply chains in developing countries.^27^ Thus, while proven practices have been well validated and remain robust methods for improving supply chain functions, the need for continuous development of supply chain expertise and the deployment of innovative, promising practices remain the solutions to underperforming supply chain systems in LMICs. Whereas the cases presented may not have deployed entirely untried methods, we have considered the solutions promising as they are being deployed in different sociocultural contexts.

The cases presented support the calls for innovative solutions to the challenges of underperforming supply chains in developing countries.

Useful lessons have been derived from the cases. The Ghana case adds to the evidence that with the right eLMIS, developing countries can build the supply chain capacity needed to streamline inventory management operations by automating tasks such as stock tracking, replenishment, and forecasting. The case further shows that with real-time visibility into inventory levels and consumption patterns, supply chain managers can optimize stock levels, reduce waste, and prevent stock-outs, leading to greater efficiency and substantial savings for the system. The EPSS case demonstrates the power of stakeholder ownership in the implementation and sustainability of interventions, while AFRiSC provides an additional viable tool for improving the knowledge and skills of vaccine supply chain agents and logisticians.

## Notes

**Peer Reviewed**

**First Published Online:** September 4, 2024.
